# Histological reclassification, histochemical characterization and c-kit immunoexpression in renal cell carcinoma

**DOI:** 10.4103/0970-1591.42616

**Published:** 2008

**Authors:** P. R. Rekha, S. Rajendiran, Shalinee Rao, Sunil Shroff, Leena D. Joseph, D. Prathiba

**Affiliations:** Department of Pathology, Sri Ramachandra Medical College and Research Institute, Porur, Chennai - 600 116, India; 2 Department of Urology, Sri Ramachandra Medical College and Research Institute, Porur, Chennai - 600 116, India

**Keywords:** C-kit, Hale's colloidal iron, renal cell carcinoma

## Abstract

**Objectives:**

Renal cell carcinoma is the most lethal of all urologic malignancies. Several parameters such as histological subtype, nuclear grade and TNM staging help in determining the prognosis and treatment options. A newer therapeutic modality has been suggested based on expression of c-kit antigen by the tumor cells. This study was designed to evaluate various histological parameters and correlate them with c-kit expression.

**Materials and Methods:**

The study was done on 40 consecutive cases of renal epithelial tumors. Histological sections were reviewed and reclassified according to WHO (2004) classification and nuclear grade assessed. Hale's colloidal iron stain was done to identify the chromophobe variant. Immunostaining with c-kit was done and its expression was studied. The results were correlated and statistical significance was assessed.

**Results:**

The age range was 31-81 years, with a male to female ratio of 2:1. Seventy per cent of the cases were clear cell RCC (ClRCC), 17.5% were chromophobe type, 7.5% were papillary RCCs and 5% cases were oncocytomas. Fuhrman nuclear grading revealed 60.5% cases to be of low grade and 39.5% high grade. Hale's colloidal iron staining was positive in chromophobe RCC and oncocytomas, while it was negative in ClRCC. Immunostaining with c-kit was positive only in oncocytomas.

**Conclusions:**

Clear cell RCC was the most common histological subtype of RCC. Clear cell RCC known to have a poor prognosis, showed a statistically significant higher nuclear grade than chromophobe and papillary RCCs which have a better prognosis. Hale's colloidal iron staining was extremely useful in distinguishing chromophobe RCC and oncocytoma from the granular cell variant of clear RCC. Our study revealed c-kit negativity in all RCC. As Imatinib could be ineffective in such tumors, its clinical activity has to be carefully assessed in such tumors through further studies.

## INTRODUCTION

Renal cell carcinoma (RCC) is the third most common urological malignancy following prostate and urinary bladder malignancies. It accounts for 3% of all malignancies. Renal cell carcinoma has the worst prognosis among all urologic malignancies.

A variety of distinct histological patterns are recognized in RCC which has led to the classification of RCC into various histological subtypes. Several studies have shown that these subtypes have distinct molecular, clinical and prognostic features. Since the therapeutic options vary depending on the histological subtypes, it becomes essential to recognize them precisely. Chromophobe renal cell carcinomas (ChRCC) are reported to have a better prognosis and a lower tendency to metastasize, though they can grow to a large size. Hale's colloidal iron is a special histochemical stain that positively stains chromophobe variant and therefore aids in distinguishing ChRCC from other subtypes of RCC.[1] The positivity in ChRCC with Hale's colloidal iron stain is due to the presence of microvesicles having free acidic groups that gets bound to colloidal iron.

Histological grading is also one of the major parameters that conveys prognosis.[2] Various grading systems have been used in the past and Fuhrman nuclear grading is one of the most widely used systems.[3]

Recent recognition of certain molecular markers such as c-kit expression in the tumor cells has led to the introduction of newer therapeutic modalities. The proto-oncogene c-kit encodes a surface membrane tyrosine kinase receptor composed of 5 immunoglobulin-like extracellular domains and a tyrosine kinase domain. Stem cell factor has been identified as the natural ligand of kit. Interaction of c-kit receptors and its ligand results in activation of the kinase domain. The consequent phosphorylation of tyrosine residue leads to activation of signal transduction pathways involved in proliferation, apoptosis and tumorigenesis. The drug Imatinib mesylate (STI-571) is a tyrosine kinase inhibitor which is used as a targeted therapy in several tumors exhibiting c-kit overexpression. Studies on c-kit expression in RCC have shown varying results. Yamazaki *et al*. have reported overexpression of c-kit antigen exclusively in ChRCC whereas Sengupta *et al*. found it to be rare.[4,5]

With this background, our study was designed to determine the Fuhrman nuclear grading and c-kit (CD 117) expression in different histological subtypes of RCC and also to evaluate the role of Hale's colloidal iron staining pattern in histochemical categorization.

## MATERIALS AND METHODS

Forty consecutive cases of renal epithelial neoplasms, retrieved from the surgical pathology files of the Department of Pathology, Sri Ramachandra Medical College and Research Institute during 2001-2005 were included in our study. Formalin-fixed, paraffin-embedded Hematoxylin-Eosin stained sections from all the cases were reviewed by two pathologists and the morphological diagnosis was made based on WHO-2004 classification of renal epithelial neoplasms. Nuclear grade for all histological subtypes except oncocytoma was assessed using the standardized criteria as mentioned in Fuhrman nuclear grading system by using Nikon ECLIPSE E600 microscope. We graded the RCC in our study into four grades. Fuhrman Grades I-IV are delineated based on the presence of nucleolus, size and the magnification at which the nucleus can be observed.

One section each from all the 40 cases was stained by Hale's colloidal iron technique. Wherever possible, blocks containing normal renal cortex along with the tumor were selected for staining so that the glomerular mesangium which stains blue, serves as a positive internal control. An already proven case of ChRCC, positive for Hale's colloidal iron stain was used as a positive control in cases where normal renal cortex was not included in the tumor sections. Perl's stain was performed on all cases simultaneously to avoid positive nonspecific staining for hemosiderin pigment.

Immunohistochemical analysis was performed on all 40 cases using five-micron-thick, formalin-fixed, paraffin-embedded sections. Polyclonal antibody directed against c-kit (polyclonal rabbit antihuman c-kit, A 4502 affinity isolated, Dakocytomation Denmark A/S) at a dilution of 1:400 was used.

Statistical analysis of chi-square test was done using statistical software SPSS 10. The histological subtype was correlated with Fuhrman nuclear grading, Hale's colloidal iron stain and c-kit immunostain positivity.

## RESULTS

Of the 40 cases of renal epithelial neoplasms studied, 38 were RCCs and two were oncocytomas. In our study the age of patients with RCC ranged from 31 to 81 years with a median of 54.5 years. Fifty-three per cent of tumors occurred between 40 and 60 years, followed by 31% in the age group of 60-80 years. The incidence below 40 years and above 80 years was 8%. Sixty-seven per cent of patients were males and 33% were females with male to female ratio of 2:1.

The reclassification according to WHO histological classification of renal tumors 2004 showed the predominant subtype to be clear cell RCC (ClRCC), comprising 70% of the total cases studied [Table 1].

**Table 1:** Distribution of cases in subtypes of renal cell carcinoma

Subtype	Number of cases (%)
Clear cell RCC	28 (70)
Pure clear cell RCC	17 (42.5)
Eosinophilic variant of clear cell RCC	6 (15)
Sarcomatoid differentiation	5 (12.5)
Chromophobe RCC	7 (17.5)
Sarcomatoid differentiation	1 (2.5)
Papillary RCC	3 (7.5)
Oncocytoma	2 (5)

RCC - Renal cell carcinoma

**Figure 1:**
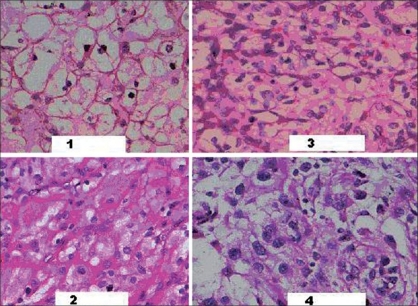
Furhman nuclear grades 1 to 4( H& E X200)

A study on Fuhrman nuclear grading was done on all 38 cases of RCC. Most of the cases belonged to Grade I and Grade II category [Table 2]. Clear cell variant of RCC showed a varied nuclear grading pattern [Figure 1]. Our study found ClRCC to have a higher nuclear grade compared to ChRCC [Table 2].

**Table 2:** Fuhrman nuclear grading in subtypes of renal cell carcinoma

	Clear cell RCC	Chromophobe RCC	Papillary RCC
Grade I	5 (12.5)	3 (7.5)	0 (0)
Grade II	12 (30)	3 (7.5)	0 (0)
Grade III	5 (12.5)	0 (0)	3 (7.5)
Grade IV	6 (15)	1 (2.5)	0 (0)

Figures in parenthesis indicate percentage, RCC - Renal cell carcinoma

**Figure 2:**
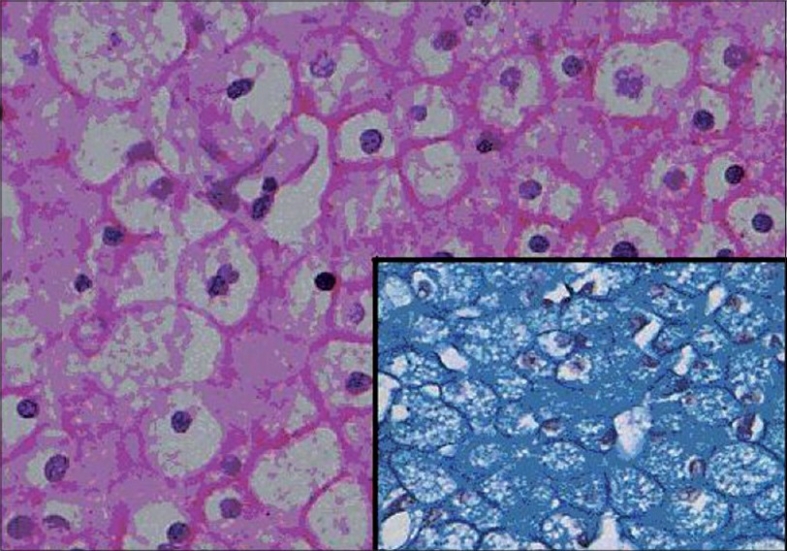
Tumor cells of Chromophobe Renal cell carcinoma showing presence of perinuclear halo (H&E X 200), inset shows positive Hale's colloidal iron staining

Hale's colloidal iron stain showed specific positivity in ChRCC and oncocytoma, however, the staining pattern was different [Figure 2]. All seven cases of ChRCC showed strong staining that was distributed diffusely throughout the cytoplasm and was characterized by a meshwork-like strong reticular positivity. The reticular pattern was better visualized at high-power examination. Among the two cases of renal oncocytoma studied, one case showed diffuse fine dust-like granules and the other case showed staining more concentrated towards the luminal aspect of the cell.

All 28 cases of Cl RCC including eosinophilic variant and those with sarcomatoid change were negative for Hale's colloidal staining [Table 3]. Although three cases of papillary RCC yielded strong, coarse, droplet positivity, most of these areas with positivity correlated with the positive staining of Perl's reaction for hemosiderin. Hence these cases were considered negative for Hale's colloidal iron. This difference in staining pattern observed between oncocytoma, ChRCC and other types of RCC was statistically significant (*P* < 0.001).

**Table 3:** Hale's colloidal iron in renal epithelial neoplasms

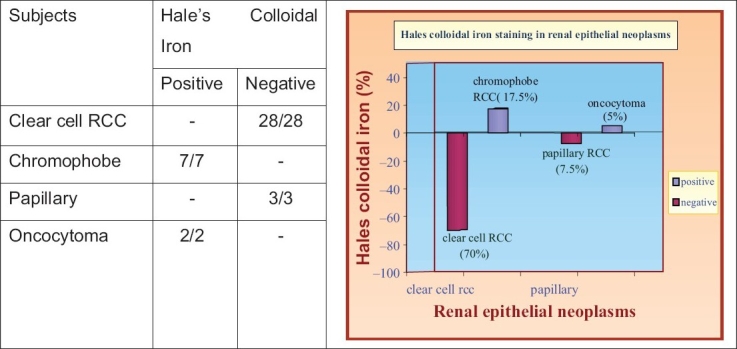

Immunostain with c-kit was positive in oncocytomas only. All the other subtypes were negative. The proximal convoluted tubules in the normal cortex adjacent to the tumor also showed c-kit positivity. Two cases of oncocytoma which were immunoreactive for c-kit showed different pattern of staining. One showed a moderate cytoplasmic and membrane positivity in focal areas of tumor and the other showed pure membrane positivity throughout the tumor. Mast cells in the stroma of PRCC showed positivity with c-kit. Statistical analysis showed a significant difference (*P* < 0.001) in staining pattern between oncocytoma and RCC.

## DISCUSSION

RCC comprises a heterogeneous group of neoplasms arising from different parts of the nephron. Previous studies have shown that the histological subtypes of RCC are genetically and biologically different. Hence we need to identify them specifically. Clear cell RCC was the most common subtype and papillary RCC was least common in our study. This was in concordance with the previous studies in the western literature.[6,7] We did not find collecting duct carcinoma and medullary carcinoma, which form only 0-1% of all RCCs as reported in the literature.[6,8,9] Sarcomatoid differentiation was seen in 15% of the total cases, which was slightly higher than the earlier report.[10] Sarcomatoid differentiation was more common in ClRCC (17.8%) followed by ChRCC (14.2%). Sarcomatoid RCC is known to be aggressive with a high potential for distant metastases. The presence of sarcomatoid component portends a poorer prognosis irrespective of the basic histological subtype as reported in the literature with patient survival ranging from nine months to one to two years.[4,11]

While careful examination of hematoxylin and eosin (H and E) stained sections of a well-sampled tumor will allow a diagnosis in the majority of cases, some renal tumors can show overlapping morphologic features, requiring the use of ancillary methods such as Hale's colloidal iron stain to reach a definitive diagnosis. Positive staining with Hale's colloidal iron stain is considered a diagnostic feature for ChRCC and has been used as a discriminating feature to differentiate it from other renal tumors. Hale's colloidal iron stain has been identified to be useful in distinguishing the eosinophilic variant of ChRCC from granular cell variant of ClRCC and oncocytoma.[1]

Though all ChRCCs and oncocytomas showed positivity, the staining pattern was different in these two types. A diffuse, strong, reticular positivity was noted in ChRCC whereas oncocytoma showed either a strong luminal staining pattern or diffuse, fine, dust-like granules. This difference in the staining pattern between ChRCC/oncocytoma and other subtypes of RCC was statistically significant *P* < 0.001 (99.9%). Tickoo *et al*. and Delong *et al.*, also demonstrated in their study that this stain is of great value in identifying ChRCC.[1,12] They found a similar staining pattern as seen in our study. All the 28 cases of ClRCC including its eosinophilic variant in our study showed no staining or only focal coarse droplet staining. The basis for focal positive staining of non-chromophobe RCC is difficult to explain but could be due to the presence of small number of microvesicles.[1] Considering the marked difference in patient survival rates in different histological subtypes it is mandatory to identify precisely the specific subtype.[6–8]

Despite the fact that ClRCC is known to have a poorer prognosis, in our study we found that the majority of them expressed a lower nuclear grade. Similar findings were also noticed in a few of the previous studies.[4,13] However, ChRCC which was expected to have a better prognosis did show a lower nuclear grade. Almost two-thirds of our ClRCC were of low grade. Earlier studies have shown varying grades among ClRCC.[2,9] In our study all the PRCC were Grade III tumors (100%), a very high figure in comparison to findings reported in the previous literature.[6,13] This difference in grade could be due to the small sample size in our study and needs confirmation by including more cases. Cancer-specific survival probabilities are extremely variable for RCC.[2] By Furhman grading the five-year survival rate is 65% in Grade I, 30% in Grade II, 32% in Grade III and 10% in Grade IV.[2]

Since RCC responds poorly to chemotherapy and radiotherapy, there is a need for alternative means of treatment especially in patients with metastasis. With the advent of drugs targeting c-kit positive tumors, expression of c-kit in RCC needs to be studied in detail to evaluate its usefulness. Recently, there has been an increasing interest in the study of c-kit expression on different neoplasms due to the therapeutic introduction of STI 571 (imatinib mesylate) an inhibitor of tyrosine kinase. Various studies have demonstrated c-kit expression in renal tumors which could be targeted for treatment using imatinib mesylate.[4] This could be more useful in the treatment of aggressive and metastatic tumors which do not have any specific and effective treatment since imatinib mesylate is a relatively non-toxic therapeutic agent.

We observed that none of the 38 cases of RCC studied showed positivity for c-kit. There were two oncocytomas identified in our study which showed membrane positivity with c-kit.

The review of the literature reveals variable findings in different studies and no consistent pattern of positivity was noted for c-kit expression in RCC. Wang *et al*. and Anna Petit found c-kit immunostaining helpful in distinguishing the granular cell variant of ClRCC which was c-kit negative, from ChRCC and oncocytoma which was positive.[14,15] Yamazaki *et al.*, included gene analysis in addition to c-kit and concluded that c-kit expression is a useful marker and would be of therapeutic value in ChRCC.[4] Positivity for c-kit in a small number of ClRCC cases was also reported by Miliaras *et al*.[16] All PRCC cases and sarcomatoid areas also stained negative for c-kit in our series as against the findings of other authors in the literature.[9,17] Sengupta *et al.*, studied a significant number of RCC and concluded that imatinib mesylate therapy is unlikely to be effective as c-kit expression was rare in high-grade RCC.[5] Only oncocytomas which are benign showed positivity for c-kit. We propose that c-kit expression may not be a consistent feature and useful marker in distinguishing ClRCC from ChRCC.

Oncocytomas and ChRCC are thought to originate from the intercalated cells of renal collecting tubule. Miliaras *et al.*, showed that intercalated cells of renal collecting tubules are CD117-negative whereas proximal convoluted tubules (PCT) are CD117-positive.[16] These findings imply that a mechanism of c-kit activation may be involved in oncocytoma and ChRCC tumorigenesis and conversely c-kit inactivation may be implicated in c-kit-negative renal epithelial tumors. A more detailed and large scale study is needed to clarify whether c-kit expression in renal tumors results from c-kit mutation or represents an epigenetic phenomenon.

Renal cell carcinoma is an aggressive tumor resistant to chemotherapy or radiation. Metastatic RCC earlier treated with the cytokines interferon alpha (IFNα) or interleukin 2 (IL-2) demonstrated low rates of efficacy along with severe infusion-related adverse reactions.[18] A newer therapeutic modality in these patients is now available as receptor tyrosine kinase (RTK) inhibitors. However, response to tyrosine kinase inhibitor therapy in c-kit-positive RCC showed variable results. In a Phase II trial done by Vuky *et al.*, no or partial response was observed in 12 of 14 patients treated with imatinib in c-kit-positive RCC.[19] As compared with interferon alfa, temsirolimus (a specific inhibitor of the mammalian target of rapamycin kinase), improved overall survival among patients with metastatic RCC.[20] On the other hand, in two separate Phase II studies treated with sunitinib the response rate was approximately 40% in patients who had already failed cytokine therapy.[18]

To conclude, Hale's colloidal iron stain was extremely useful in distinguishing chromophobe RCC and oncocytoma from the granular cell variant of ClRCC. Our study revealed c-kit negativity in all RCC. As imatinib could be ineffective in such tumors, its clinical activity has to be carefully assessed in such tumors through further studies.
